# CB1 receptor blockade ameliorates hepatic fat infiltration and inflammation and increases Nrf2-AMPK pathway in a rat model of severely uncontrolled diabetes

**DOI:** 10.1371/journal.pone.0206152

**Published:** 2018-10-26

**Authors:** Eugene Chang, Dae-Hee Kim, Hyekyung Yang, Da Hyun Lee, Soo Han Bae, Cheol-Young Park

**Affiliations:** 1 Department of Nutritional Science and Food Management, Ewha Womans University, Seoul, Seoul, Republic of Korea; 2 Medical Research Institute, Kangbuk Samsung Hospital, Sungkyunkwan University School of Medicine, Seoul, Republic of Korea; 3 Severance Biomedical Science Institute, Yonsei Biomedical Research Institute, Yonsei University College of Medicine, Seoul, Republic of Korea; 4 Division of Endocrinology and Metabolism, Department of Internal Medicine, Kangbuk Samsung Hospital, Sungkyunkwan University School of Medicine, Seoul, Republic of Korea; Medizinische Fakultat der RWTH Aachen, GERMANY

## Abstract

Previous studies have shown that the CB1 receptor antagonist reverses steatohepatitis and its related features of metabolic syndrome, such as obesity and type 2 diabetes. However, the beneficial effects of CB1 receptor blockade on hepatic steatosis and inflammation have not been investigated independently of its effects on body weight and glycemic control. At 32 weeks of age, OLETF rats were administered with rimonabant (10 mg·kg^−1^·day^−1^) by oral gavage for 6 weeks. No significant changes in body weight, OGTT, and serum glucose were observed in spite of rimonabant-decreased food intake. Moreover, there was a significant difference between initial and final body weight, regardless of rimonabant administration, indicating that OLETF rats were severely diabetic rats. Rimonabant administration significantly decreased serum liver enzyme levels such as ALT and AST, hepatic fat accumulation, lipid peroxidation, and cell death as demonstrated by the number of TUNEL-positive cells in severely uncontrolled diabetic OLETF rats. Significant decreases in hepatic gene expression of proinflammatory cytokines (CD11b, F4/80, MCP1, and TNFα), negative inflammatory mediators (SOCS1 and SOCS3), and fibrosis-related proteins (TGFβ, collagen 1, and TIMP1) were found in rimonabant-treated OLETF rats. Six-week administration of rimonabant significantly upregulated mRNA levels of CPT1α and PPARα related to β-oxidation. Moreover, significant increases in Nrf2 gene expression and its downstream genes, NQO1, GSAT, HO-1, and TXNRD1 along with increased AMPK phosphorylation were noted in uncontrolled diabetic rats treated with rimonabant. The observed potent inhibitory effects of CB1 receptor blockade on hepatic fat infiltration and cellular death in severely uncontrolled diabetic rats indicate that CB1 receptor is a possible therapeutic target. Increased Nrf2 and AMPK phosphorylation may play a role in the mechanism of rimonabant action.

## Introduction

Nonalcoholic fatty liver disease (NAFLD) encompasses a wide range of diseases from simple steatosis (aberrant hepatic lipid accumulation) to hepatic inflammation, hepatocellular ballooning, hepatic injury and fibrosis, and cellular death, all of which are referred to as nonalcoholic steatohepatitis (NASH). NASH results in cirrhosis and hepatocellular carcinoma [1, 2]. Advanced fibrosis has been reported in 5–7% of asymptomatic individuals with type 2 diabetes [3, 4]. Individuals with severe diabetes are more likely to have more severe NAFLD with hepatic inflammation and fibrosis [5, 6]. Still, the pathogenesis of NASH is not fully understood. In addition, there is no available pharmacotherapy to fully reverse and prevent NASH. Thus, it is important to explore possible therapeutic strategies for NASH.

Growing evidence indicates that the endocannabinoid system is a key regulator of food intake, glucose and lipid metabolism, and energy balance [7, 8] and becomes over-activated in obesity and type 2 diabetes mellitus [9, 10]. In the endocannabinoid system, endogenous arachidonic acid-derived mediators, endocannabinoids, and cannabinoid 1 (CB1) receptor have been detected in the liver. Therefore, the liver has been considered as a primary tissue for endocannabinoid-mediated metabolic dysfunction [11, 12]. Indeed, the CB1 receptor activation increases *de novo* hepatic lipogenic gene expression and decreases the activity of carnitine palmitoyltransferase-1 (CPT1) [11, 13]. In addition, the endocannabinoid system via CB1 receptor contributes to hepatic inflammation, fibrosis, cellular death, and hepatocellular carcinoma initiation [14–16]. Thus, novel therapeutics have been investigated to block CB1 receptor activity in conditions that predispose to liver fibrosis. However, the molecular mechanisms by which the CB1 receptor antagonist affects hepatic inflammation and fibrosis have not been fully determined apart from its effects on weight loss and hypoglycemia.

Chronic oxidative stress plays a critical role in the development of liver fibrosis, which is attributable to its close association with lipid metabolism [17]. Nuclear factor erythroid 2-related factor 2 (Nrf2, Nfe2l2), a transcription factor, serves as a major regulator of cellular defense system against oxidative stress. Upon oxidative stress, the dissociation of Nrf2 from a sequestration complex leads to Nrf2’s translocation to the nucleus, where it interacts with antioxidant-responsive elements (ARE) and activates the transcription of its target genes, such as hemeoxygenase 1 (HO-1), superoxide dismutase, glutathione peroxidase, glutathione S-transferase (GSAT), catalase, NAD(P)H quinone oxidoreductase 1 (NQO1), and thioredoxin reductase 1 (TXNRD1) [18, 19]. Several lines of evidence demonstrate that genetic deletion of Nrf2 is associated with more severe NASH [20, 21]. In addition, increased Nrf2 activity contributes to AMP-activated protein kinase (AMPK) phosphorylation in the liver [22]. AMPK, a central regulator of cellular energy homeostasis and inflammation, modulates fatty acid biosynthesis [23] and inhibits reactive oxidative stress and inflammation [24, 25]. Due to the critical roles of Nrf2 and AMPK in oxidative stress and lipid metabolism, they are potential therapeutic targets for treatment of hepatic lipid infiltration and inflammation

In previous studies, genetic and pharmacological modifications of CB1 receptors modulates the fibrogenic process [26, 27]. However, the therapeutic role of CB1 receptor blockade in severely uncontrolled diabetic rats, characterized by decreased body weight and hyperglycemia, and its underlying mechanism in relation to decreasing hepatic fat accumulation and inflammation have never been determined. Therefore, in the present study, we investigate the therapeutic efficacy of rimonabant (SR141716), a potent and selective CB1 receptor antagonist, on hepatic fat accumulation, inflammation, and death in a rat model of severely uncontrolled diabetes.

## Materials and methods

### Animal experiments

Animal housing and procedures were approved by the Animal Experiments Ethics Committee of the Sungkyunkwan University, Kangbuk Samsung Hospital. Otsuka Long-Evans Tokushima Fatty (OLETF) and age-matched Long-Evans Tokushima Otsuka (LETO) rats were purchased from Otsuka Pharmaceutical Company (Tokushima, Japan). Rats at 4 weeks of age were maintained in a temperature and humidity-controlled specific pathogen-free facility on a 12-h light/12-h dark cycle and had unrestricted access to water and a standard irradiated rodent chow diet (PicoLab Rodent Diet 20 5053, 5% wt/wt fat, Purina Mills, Richmond, IN, USA). To investigate the role of CB1 receptor blockade via rimonabant in hepatic lipid accumulation and inflammation, 20 rats at 32 weeks of age were treated with either PBS as vehicle or rimonabant (10 mg·kg^-1^·day^-1^, Sanofi-Aventis, Paris, France) by daily oral gavage for an additional 6 weeks. Pair-fed controls were given a daily amount of food equal to that consumed by rimonabant-treated counterparts over the previous 24 h period. Body weight and food intake were monitored daily. At the end of 6-week animal experiment, 38-week-old LETO and OLETF rats were fasted overnight and anesthetized with intraperitoneal Zoletil/Rompun. Blood was collected from abdominal aorta and liver tissues were dissected, immediately frozen in liquid nitrogen, and stored at −80°C until further analysis.

### Oral glucose tolerance test

An oral glucose tolerance test (OGTT) was performed after 16 h fasting. Rats received an oral administration of glucose solution (2 g/kg body weight) by stomach gavage. Blood glucose levels were measured at 0, 15, 30, 60, 90, and 120 min after the glucose challenge, using a Glucocard X-Meter (Arkray, Kyoto, Japan). The area under the curve (AUC) was calculated and the difference (ΔAUC) was reported.

### Measurement of serum and hepatic metabolic parameters

Serum glucose, alanine aminotransferase (ALT), and aspartate aminotransferase (AST) were analyzed by enzymatic enzyme methods (Sigma-Aldrich, St. Louis, MO, USA). For hepatic triglyceride (TG) quantification, lipid was extracted as described in a previous study [28]. Briefly, liver tissue was homogenized in 1 mL solution containing 5% Nonidet P-40 (NP-40) substitute (Amresco, Solon, OH, USA). The homogenates were slowly heated to 80–100°C in a water bath for 2–5 min until the NP-40 became cloudy, then cooled down to room temperature. Samples were then centrifuged for 2 min to remove any insoluble materials. Hepatic TG levels were measured by enzymatic assay (Sigma-Aldrich) and normalized to their respective protein concentrations. Lipid peroxidation (MDA) levels in the liver tissue were measured by a OxiSelect^TM^ TBARS assay kit (Cell Biolabs, San Diego, CA, USA) according to the manufacturer’s protocol, normalized to their respective protein concentrations, and expressed as fold change compared to LETO control group.

### Histological analysis and NAFLD activity score (NAS)

Dissected liver tissues were fixed with 10% neutral formalin buffer overnight, embedded in paraffin, sectioned, and stained with hematoxylin and eosin (H&E) and Masson's trichrome. Digital images were acquired with a microscope (Olympus BX51 light microscope, Tokyo, Japan). To investigate the severity of NAFLD or fibrosis, a pathologist blinded to the experiment measured the NAFLD activity score (NAS) or fibrosis score. NAS was calculated by measuring three features of NAFLD and adding up their scores: steatosis (0–3), ballooning (0–2), and inflammation (0–3) [29]. To illustrate the fibrosis stage, fibrosis score was calculated by summing up scores: no fibrosis (0), perisinusoidal or periportal fibrosis (1), perisinusoidal and portal/periportal fibrosis (2), bridging fibrosis (3), and cirrhosis (4) [30].

### TUNEL assay

The terminal deoxynucleotidyl transferase dUTP nick-end labeling (TUNEL) assay was performed using an In Situ Cell Death Detection Kit, TMR red (Roche, Indianapolis, IN, USA), according to the manufacturer’s instruction. The TUNEL assay is based on the detection of DNA fragmentation by labeling 3'-hydroxyl ends in double-stranded DNA breaks in the early stages of apoptosis. Nuclei were counterstained with 4',6-diamidino-2-phenylindole (DAPI, Sigma-Aldrich) for 10 min, and the slides were observed and visualized on a LSM700 confocal microscope (Carl Zeiss, Jena, Germany) at 800 X magnification. Results were expressed as the number of TUNEL-positive cells per microscopic field.

### RNA isolation, reverse transcription, and real-time quantitative polymerase chain reaction (RT-PCR)

Isolation of RNA from liver tissues was performed using an RNeasy Mini Kit (Invitrogen, Carlsbad, CA, USA) as described by the manufacturer’s protocol. cDNA was synthesized from isolated total RNA using a high-capacity RNA-to-cDNA Kit (Applied Biosystems, Foster City, CA, USA). The polymerase chain reaction (PCR) was performed using a LightCycler 480 Probes Master Mix and a Lightcycler 480 system (Roche). Primers used are shown in Table 1. The PCR parameters were as follows: pre-denaturation at 95°C for 10 min, followed by 45 cycles of denaturation at 95°C for 10 s and annealing/extension at 60°C for 20 s. Data were analyzed using the ΔΔCt method for relative quantification [31]. Expression of each target gene was normalized to the housekeeping genes such as glyceraldehyde-3-phosphate dehydrogenase (GAPDH) and ribosomal RNA (18S) and expressed as fold change relative to LETO control group.

**Table 1:** Primers used for RT-PCR.

Gene	GeneBank no.	Forward sequence (5’-3’)	Reverse sequence (5’-3’)	Product size (bp)
CD11b	NM_012711.1	TCAAGGTCGTTGTGACCAGT	CACAGGCAACTCCAACTGAG	74
Collagen1	NM_053304.1	CATGTTCAGCTTTGTGGACCT	GCAGCTGACTTCAGGGATGT	94
CPT1α	NM_031559.2	ACAATGGGACATTCCAGGAG	AAAGACTGGCGCTGCTCA	65
F4/80	NM_001007557.1	AGACTGGCCCCAAGAAACTC	ATAATCGCTGCTGGCTGAAT	60
GAPDH	NM_017008.4	AGCTGGTCATCAACGGGAAA	ATTTGATGTTAGCGGGATCT	63
GSAT	NM_031509.2	AGTCCTTCACTACTTCGATGGCAG	CACTTGCTGGAACATCAAACTCC	151
HO-1	NM_012580.2	CGACAGCATGTCCCAGGATT	TCGCTCTATCTCCTCTTCCAGG	184
MCP1	NM_031530.1	AGCATCCACGTGCTGTCTC	GATCATCTTGCCAGTGAATGAG	78
Nrf2	NM_031789.2	ACATCCTTTGGAGGCAAGAC	GCCTTCTCCTGTTCCTTCTG	145
NQO1	NM_017000.3	GTGAGAAGAGCCCTGATTGT	CCTGTGATGTCGTTTCTGGA	167
PPARα	NM_013196.1	TGCGGACTACCAGTACTTAGGG	GCTGGAGAGAGGGTGTCTGT	72
18S rRNA	V01270.1	GATTAGTCCCTGCCCTTTGT	GATCCCGAGGGCCTCAACTAAAC	
SOCS1	NM_145879.2	CAGCCGACAATGCGATCT	CGAGGACGAAGACGAGGAC	77
SOCS3	NM_053565.1	AATCCAGCCCCAATGGTC	GGCCTGAGGAAGAAGCCTAT	65
TGFβ	NM_021578.2	CCTGGAAAGGGCTCAACAC	CTGCCGTACACAGCAGTTCT	100
TIMP1	NM_053819.1	CAGCAAAAGGCCTTCGTAAA	TGGCTGAACAGGGAAACACT	70
TNFα	NM_012675.3	GCCCAGACCCTCACACTC	CCACTCCAGCTGCTCCTCT	99
TXNRD1	NM_001351983.1	AAGGTGACCGCTAAGTCCAC	CATTGATCTTCACGCCCACG	130

CD11b, CD11 antigen-like family member b; CPT1α, carnitine palmitoyltransferase 1α; F4/80, EGF-like module-containing mucin-like hormone receptor-like 1; GAPDH, glyceraldehyde-3-phosphate dehydrogenase; GSAT, glutathione S-transferase; HO-1, heme oxygenase 1; MCP1, CCR2 monocyte chemoattractant protein 1; Nrf2, nuclear factor erythroid 2-related factor 2; NQO1, NAD(P)H quinone oxidoreductase 1; PPARα, peroxisome proliferator-activated receptor α; SOCS1, suppressor of cytokine signaling 1; SOCS3, suppressor of cytokine signaling 3; TGFβ, transforming growth factor β; TIMP1, tissue inhibitor of metalloproteinase 1; TNFα, tumor necrosis factor α; TXNRD1, thioredoxin reductase 1

### Western blot analysis

Total protein was isolated from liver tissues by homogenization in an ice-cold lysis buffer containing 20 mM HEPES-KOH (pH 7.9), 125 mM NaCl, 10% glycerol, 0.3% Triton X-100, 1 mM EDTA, 0.5% NP-40, 10 mM β-phosphoglycerate, 1 mM Na_3_VO_4_, 5 mM NaF, 1 mM aprotinin, 1 mM phenylmethanesulfonylfluoride, and 1 mM leupeptin. After centrifugation, supernatants were collected and subjected to Western blot analysis. Equal amounts of protein were separated by electrophoresis on 12% or 14% sodium dodecyl sulfate polyacrylamide gels and transferred to polyvinylidene difluoride membrane (Millipore, Marlborough, MA, USA). Membranes were blocked in 5% nonfat dry milk in Tris-buffered saline/Tween-20 (50 mM Tris pH 7.5, 500 mM sodium chloride, and 0.05% Tween-20) for 1 h at room temperature. Membranes were incubated overnight at 4°C with the following primary antibodies: anti-β actin (AbClon, Seoul, South Korea), anti-HO-1 (Santa Cruz Biotechnology, Santa Cruz, CA, USA), NQO1 (Santa Cruz Biotechnology), anti-phospho AMPK (Cell Signaling Technology, Danvers, MA, USA), and anti-AMPK (Cell Signaling Technology). Membranes were then exposed to an anti-rabbit secondary antibody conjugated to horseradish peroxidase (Cell Signaling Technology) for 1 h at room temperature. Signals were detected by enhanced chemiluminescence lighting solution (Young In Frontier, Seoul, South Korea). Densitometry analysis was performed using ImageJ software (National Institutes of Health, Bethesda, MD, USA).

### Statistical analysis

Data are expressed as mean ± standard error of mean (SEM). Statistical differences among groups were determined by Student’s t-test for the comparison of two groups or by one-way analysis of variance (ANOVA) following Tukey multiple comparison post hoc test. Statistical significance was defined as P < 0.05 using PASW Statistics 18 (SPSS, Chicago, IL, USA).

## Results

### CB1 receptor blockade did not reduce body weight and did not improve glucose control in a rat model of severely uncontrolled diabetes

**Fig 1:**
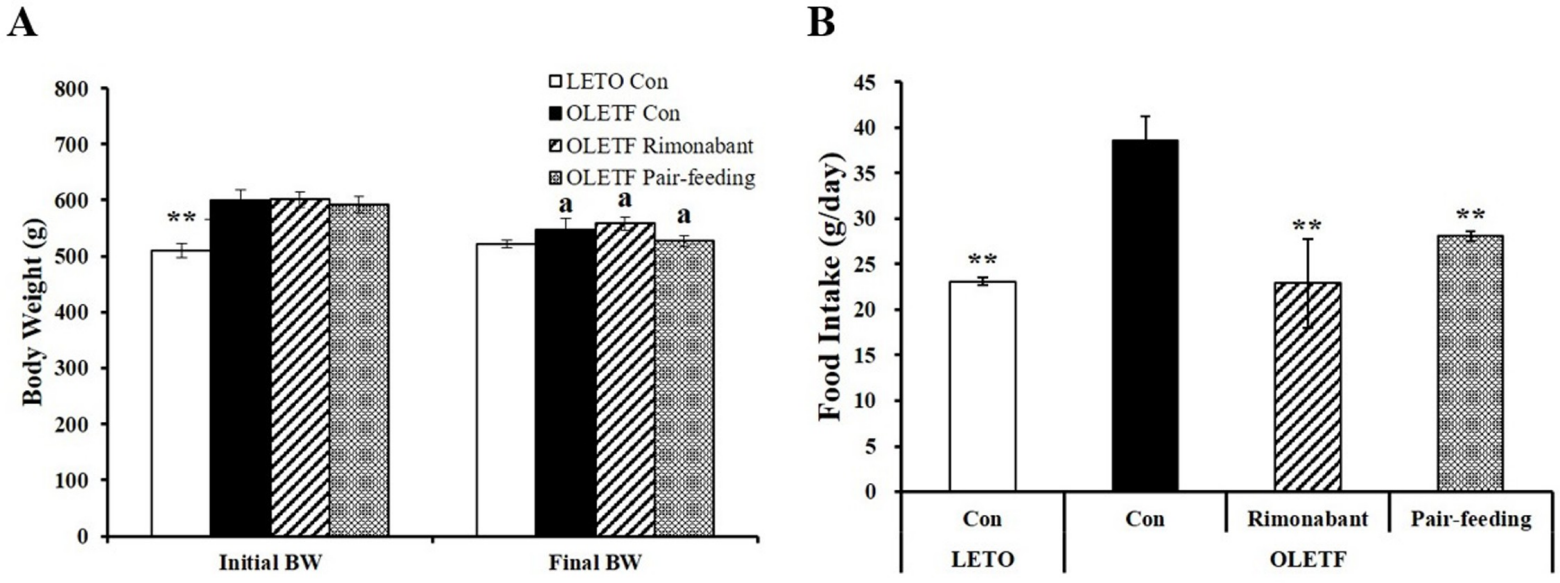
**Effects of CB1 receptor antagonist, rimonabant on body weight gain (A) and food intake (B).** LETO or OLETF rats were administrated either PBS or rimonabant (10 mg/kg/day) for 6 weeks. The pair-fed OLETF rats were given the same amount of food as their counterpart rimonabant-treated OLETF rats had consumed over the previous day. Data are expressed as mean ± SEM (n = 4–5 per group). ** P < 0.01 compared to OLETF control rats (OLETF con). a P < 0.05 compared to initial body weight.

**Fig 2:**
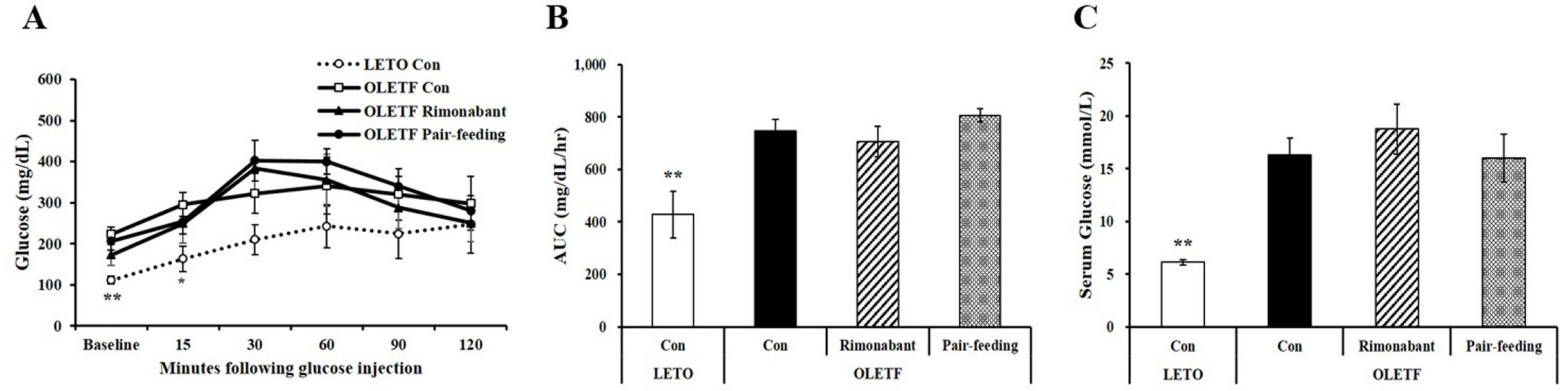
Influence of rimonabant on oral glucose tolerance test (OGTT) and fasting serum glucose concentrations. (A) An OGTT was performed before the 6 weeks of administration, and area under the curve (AUC) is shown in (B). Serum fasting glucose level (C). Data are represented as mean ± SEM (n = 4–5 per group). *P < 0.05; **P < 0.01 compared to OLETF control rats (OLETF con).

At 32 weeks of age, initial body weights of OLETF groups (OLETF con, OLETF Rimonabant, and OLETF Pair-feeding) were not statistically different (Fig 1A). Six-week administration of rimonabant did not change final body weight, even though rimonabant-treated rats significantly decreased their food intake compared to OLETF control rats (Fig 1A and 1B). Regardless of rimonabant administration, a significant difference was observed between initial (597.7 ± 8.38 g) and final body weights (544.2 ± 8.61 g) in OLETF control animals fed a chow diet (P < 0.05). Based on decreased final body weight, 38-week-old OLETF rats can be considered to be a rat model of severely uncontrolled diabetes. In addition, CB1 receptor blockade did not improve glycemic control, as indicated by OGTT, AUC, and fasting serum glucose concentration (Fig 2) in severely uncontrolled diabetic OLETF rats.

### Rimonabant ameliorated hepatic fat accumulation without changing body weight and glycemic control in uncontrolled diabetic OLETF rats

**Fig 3:**
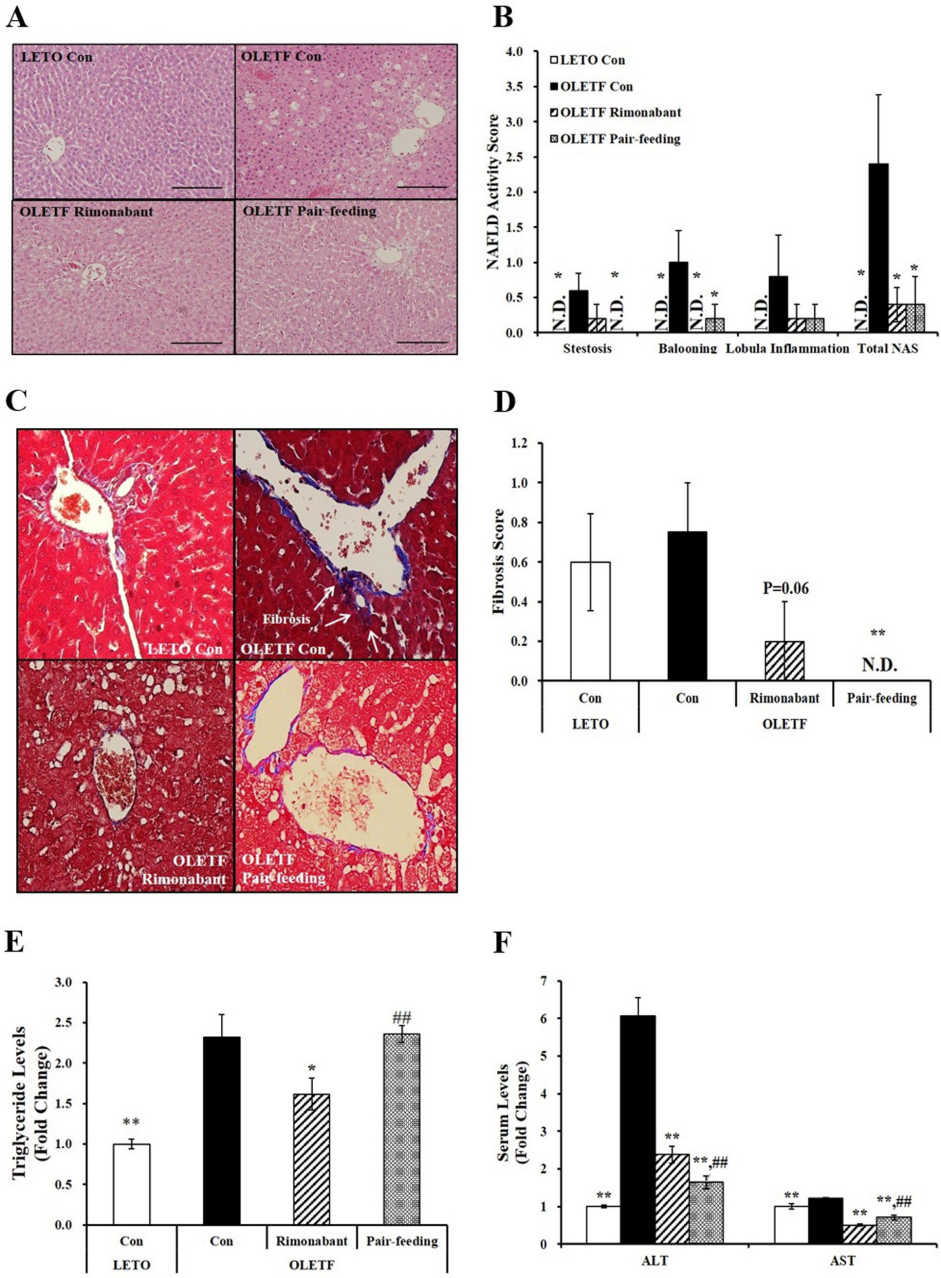
Rimonabant improves hepatic steatosis in OLETF rats. (A) Representative hematoxylin and eosin-stained liver sections (scale bars, 200 μm; magnification, 200 X). (B) NAFLD activity score. Representative images of Masson’s trichrome staining (C; scale bars, 100 μm; magnification, 400 X) and fibrosis score (D). Hepatic triglyceride concentrations (E) and serum levels of ALT and AST (F) were expressed as fold change compared to LETO control group (LETO Con). Data are expressed as mean ± SEM (n = 4–5 per group). *P < 0.05, **P < 0.01 compared to OLETF control rats (OLETF con). ##P < 0.01 compared to rimonabant-treated OLETF rats (OLETF rimonabant). N.D., Not detected; NAFLD, Nonalcoholic fatty liver disease; NAS, NAFLD activity score.

Next, we investigated whether CB1 receptor blockade can improve hepatic steatosis in conditions of unexpected weight loss and uncontrolled hyperglycemia, which are features of severe uncontrolled diabetes. At 38 weeks of age, OLETF control rats showed larger lipid droplets and ballooning in hepatocytes than age-matched LETO controls; these features were attenuated in rimonabant-treated OLETF rats (Fig 3A). Similar to these features, there was a significant reduction of NAFLD activity score (NAS) in rimonabant-treated OLETF rats (Fig 3B). In addition, Masson’s trichrome staining showed little or mild fibrosis in 38 week- old OLETF rats. Without statistical difference, an increasing trend of fibrosis score in the liver tissues from OLETF rats was reversed by 6-week rimonabant administration (P = 0.06, Fig 3C and 3D). Moreover, hepatic TG concentrations were significantly decreased in rimonabant-treated OLETF rats (Fig 3E). In addition, pair-feeding did not lead to a significant reduction in hepatic TG levels (Fig 3E), indicating that decreased hepatic fat accumulation in rimonabant-treated rats might be independent of rimonabant-induced food reduction in severely uncontrolled diabetic OLETF rats. Furthermore, OLETF control rats showed significantly higher serum liver enzyme markers such as ALT (39.0 ± 3.2 IU/L) and AST (427.5 ± 30.6 IU/L) compared to LETO control animals (ALT, 6.4 ± 0.2; AST, 353.7 ± 74.0 IU/L), which were markedly reduced by rimonabant (Fig 3F). Therefore, 38-week-old severely uncontrolled diabetic OLETF rats might be a rodent model of early stage of NASH representing hepatic steatosis as demonstrated by fat accumulation and increased serum ALT and AST concentrations, and little fibrosis. Six-week rimonabant administration alleviated hepatic fat deposition and fibrosis in OLETF rats in the conditions of severely uncontrolled diabetes and early stage of NASH.

### CB1 receptor blockade decreases lipid peroxidation and cell death in liver tissues of OLETF rats

**Fig 4:**
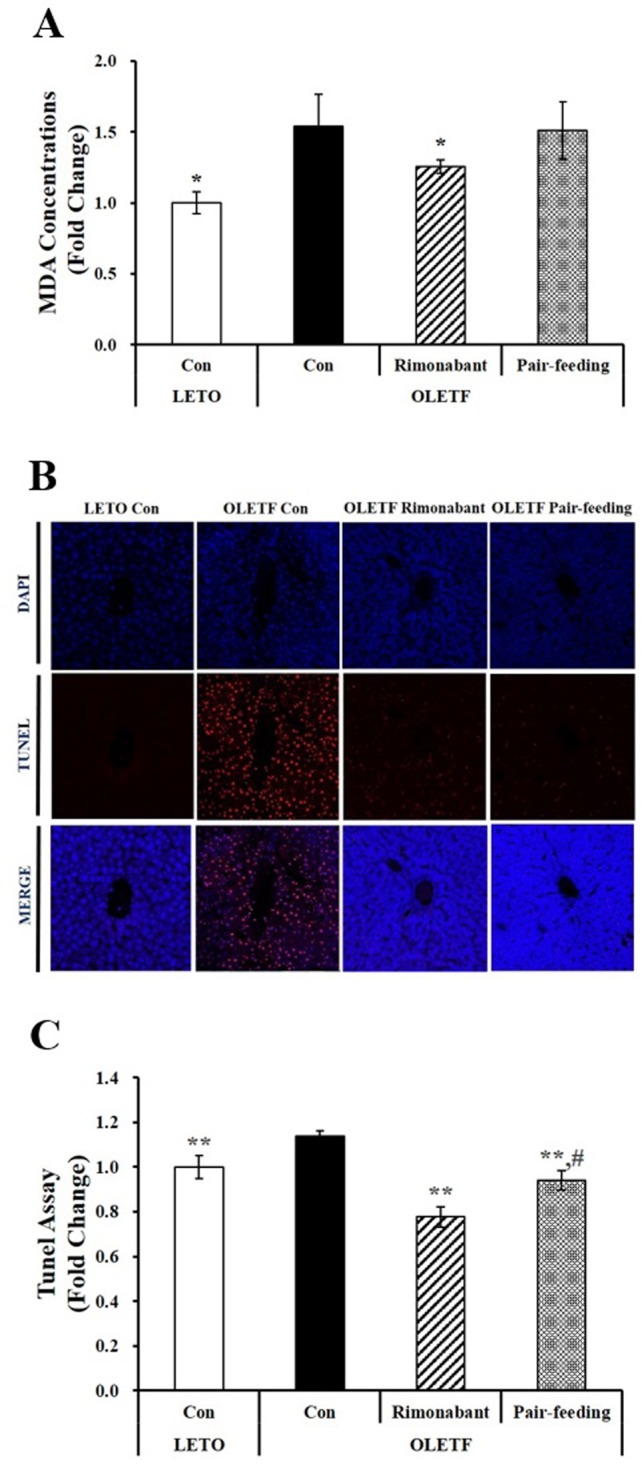
Cell apoptosis in the livers of OLETF rats administered either PBS or rimonabant for 6 weeks. (A) MDA concentrations were normalized to their respective protein concentrations and expressed as fold change compared to LETO control group (LETO Con). (B) TUNEL-positive apoptotic hepatocytes showing red under a confocal microscope (scale bars, 50 μm; magnification, 800 X). (C) Semi-quantification of TUNEL-positive cells from four randomly selected fields were normalized to LETO rats (LETO Con). Results are represented as mean ± SEM (n = 4–5 per group). *P < 0.05, **P < 0.01 compared to OLETF control rats (OLETF con). #P < 0.05 compared to rimonabant-treated OLETF rats (OLETF rimonabant).

To investigate the involvement of CB1 receptor in hepatic oxidative stress and damage, we next measured the amount of hepatocellular lipid peroxidation and cell death present in OLETF rats having weight depletion and uncontrolled hyperglycemia. A marker of oxidative stress as detected by MDA levels was significantly increased in 38-week-old OLETF rats fed with a chow diet, which was reversed after 6-week rimonabant administration (P < 0.05) (Fig 4A). In addition, increased TUNEL-positive cells was observed in severely uncontrolled diabetic OLETF control rats (Fig 4B). The number of TUNEL-positive cells was significantly higher in OLETF control group compared to rimonabant-treated OLETF group (Fig 4C). CB1 receptor blockade significantly decreased the number of TUNEL-positive cells (Fig 4B and 4C), suggesting a beneficial effect of rimonabant on liver death in OLETF rats with severely uncontrolled diabetes.

### Rimonabant modulates hepatic fatty acid oxidation, inflammatory, and fibrosis gene markers in OLETF rats

**Fig 5:**
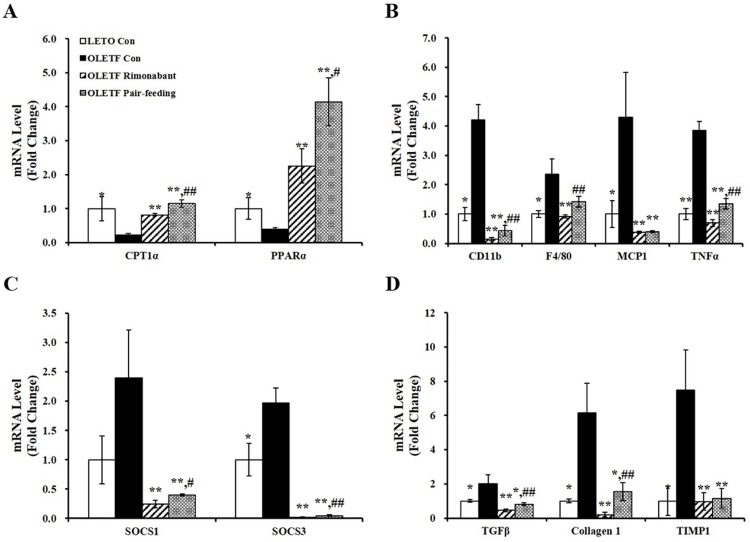
Rimonabant mediates gene expression responsible for lipid catabolism and cell death in the livers of OLETF rats. mRNA levels involved in fatty acid oxidation (A), proinflammatory cytokines (B), negative inflammatory markers (C), and fibrosis (D) were analyzed by RT-PCR and normalized for all samples to GAPDH level. The value of each bar is represented as mean ± SEM (n = 4–5 per group). *P < 0.05; **P < 0.01 compared to OLETF control rats (OLETF con). #P < 0.05; ##P < 0.01 compared to rimonabant-treated OLETF rats (OLETF rimonabant).

We evaluated whether rimonabant significantly reduced hepatic TG levels by regulating genes involved in fatty acid oxidation. mRNA levels of CPT1α and peroxisome proliferator-activated receptor α (PPARα), which are involved in β-oxidation, were significantly upregulated by 6 weeks of rimonabant administration in severely uncontrolled diabetic OLETF rats (Fig 5A). Next, to investigate the inhibitory effects of rimonabant on liver fibrosis and cellular death, gene expression involved in inflammation and fibrosis was measured. CB1 receptor blockade significantly downregulated proinflammatory CD11 antigen-like family member b (CD11b), EGF-like module-containing mucin-like hormone receptor-like 1 (F4/80), CCR2 monocyte chemoattractant protein 1 (MCP1), and tumor necrosis factor α (TNFα) gene expression (Fig 5B). Consistent with significant reductions in mRNA abundance related to inflammation, gene levels of negative inflammatory mediators [32, 33] such as suppressor of cytokine signaling 1 (SOCS1) and SOCS3, were significantly downregulated by rimonabant (Fig 5C). In addition, rimonabant administration significantly suppressed hepatic profibrogenic transforming growth factor β (TGFβ), collagen type 1, and tissue inhibitor of metalloproteinase 1 (TIMP1) gene levels (Fig 5D).

### Rimonabant administration increases Nrf2, its downstream gene expression, and AMPK phosphorylation

**Fig 6:**
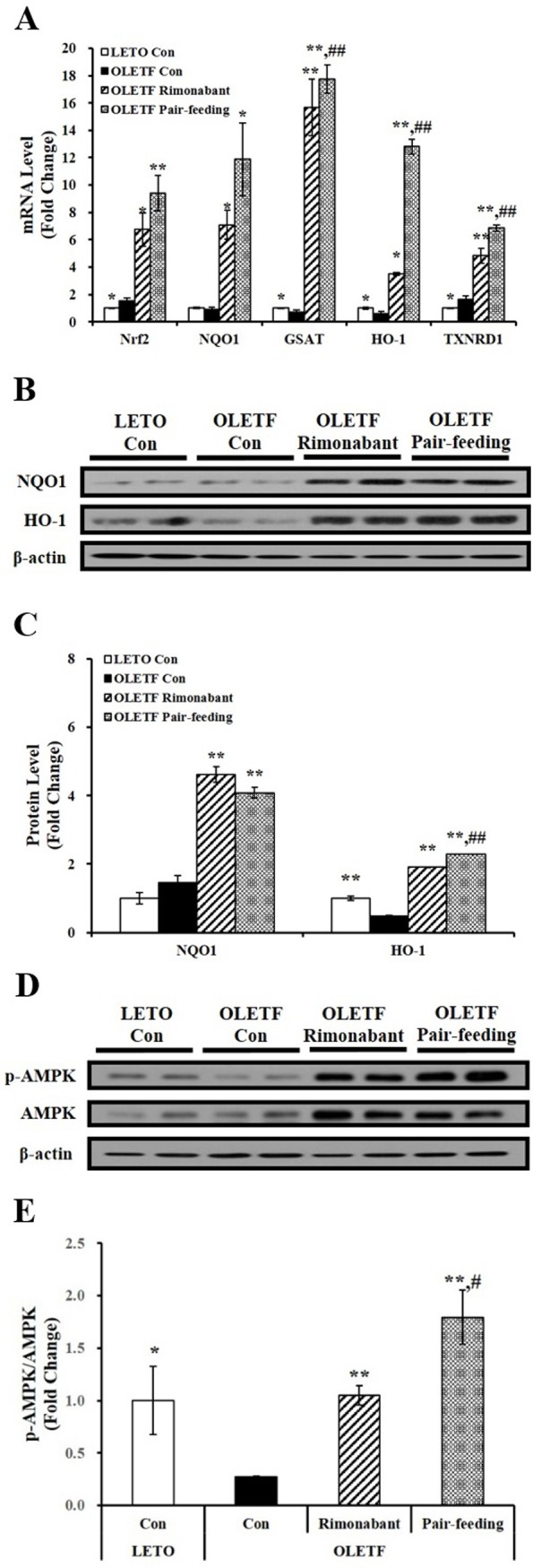
Effects of rimonabant on Nrf2 and its downstream gene expression and AMPK phosphorylation in rat livers. (A) Hepatic Nrf2 and antioxidant-responsive element (ARE)-mediated NQO1, HO-1, GSTA, and TRNRD1 gene expression were determined by RT-PCR, normalized for all samples to ribosomal RNA (18S) level, and expressed as fold change compared to LETO control rats (LETO Con). Representative western blots for NQO1, HO-1, and β-actin (B) and p-AMPK, AMPK, and β-actin (D). The density of signal was quantified and normalized by β-actin (C) or AMPK (E). Data are expressed as mean ± SEM (n = 4–5 per group). *P < 0.05; **P < 0.01 compared to OLETF control rats (OLETF con). #P < 0.05; ##P < 0.01 compared to rimonabant-treated OLETF rats (OLETF rimonabant).

In relation to hepatic inflammation, mRNA levels of hepatic Nrf2 and its downstream genes such as NQO1, HO-1, GSTA, and TXNRD1, regulators of a cellular defense system against oxidative stress, were determined in the present study. Rimonabant-treated OLETF rats showed significantly increased hepatic mRNA levels of Nrf2 and NQO1, compared to OLETF control animals (Fig 6A). In accordance with increased gene expression, hepatic protein levels of NQO1 and HO-1 also significantly upregulated by 6-week rimonabant administration in OLETF rats (Fig 6B and 6C). Next, we examined whether CB1 receptor antagonist affects AMPK activation in addition to gene expression of Nrf2 and its downstream effectors. As shown in Fig 6D and 6E, there was a further stimulatory effect of CB1 receptor blockade on liver AMPK activation, as shown as AMPK phosphorylation.

## Discussion

CB1 receptor blockade via rimonabant has been shown to reverse hepatic lipid infiltration [27, 34] and steatohepatitis [27, 35] by modulating hepatic fat synthesis, inflammation, and cell death [13, 15, 27]. Thus, although rimonabant has been withdrawn from the market because of an increase in psychiatric disorders [36], it is still important to determine the role of CB1 receptor in the pathogenesis of hepatic steatosis and the progression of NASH, and to investigate its underlying mechanism, especially independently of CB1-receptor-mediated weight loss and glycemic control. In the present study, we have shown that the administration of CB1 receptor antagonist, rimonabant significantly improved hepatic steatosis and inflammation, which was accompanied by an increase in the Nrf2 and its downstream effectors such as NQO1, HO-1, GSAT, and TXNRD1 expression, major regulators of a cellular defense system against oxidative stress, as well as AMPK phosphorylation in severely uncontrolled diabetic OLETF rats.

The relationship of endocannabinoid system via the CB1 receptor to numerous physiological processes including food intake, energy balance, and glucose metabolism has been well known [7, 37]. An overactive endocannabinoid/CB1 receptor system contributes to visceral obesity [10], obesity-related complications including type 2 diabetes [9, 38], and the pathology of fatty liver disease [39]. Inconsistent with previous studies, significant changes in weight reduction and glucose control were not observed in the present study in spite of decreased food intake in rimonabant-treated OLETF rats. In addition, we observed that significantly decreased final body weight was observed in OLETF rats at 38 weeks of age compared to initial body weight at 32 weeks of age. OLETF rats have been regarded as a strain of obese and spontaneously diabetic rats characterized by late onset of hyperglycemia, usually after 18 weeks of age [40, 41]. In addition, individuals with severe diabetes and obesity undergo weight loss without appropriate medication in uncontrolled diabetes [42]. Therefore, we speculate that 38-week-old OLETF rats fed a rodent chow diet might be in an uncontrolled severely diabetic state.

In the present study, decreased lipid accumulation and intracellular TG levels were observed in the liver tissues of rimonabant-treated OLETF rats. CB1 receptor activation leads to increased hepatic lipogenic gene expression and decreased CPT1 activity [11, 13], demonstrating the involvement of CB1 signaling in fatty acid oxidation [43]. Similar to these results, in the current study, 6-week rimonabant administration was associated with significantly increased mRNA levels of CPT1α and PPARα in the liver. In the process of fatty acid oxidation, lipid catabolism results from CPT1α-induced entry of long-chain fatty acids into mitochondria [44] and peroxisome proliferative activated receptor gamma coactivator 1α(PGC1α)-induced oxidative metabolism in cooperation with its nuclear receptor, PPARα [45]. These data suggest that CB1 receptor blockade-decreased hepatic steatosis might be associated with increased fatty acid oxidation capacity.

Oxidative stress and cellular death take part in the development and aggravation of simple steatosis and hepatic fat accumulation into NASH due to hepatocellular injury, chronic inflammation, and fibrosis [1, 2]. During chronic liver injury, hepatic macrophages release proinflammatory cytokines such as TNFα, which is followed by the infiltration of CD11b-F4/80 monocytes [46, 47]. In addition, MCP1 is involved in the monocyte/macrophage infiltration into the liver through the activation of C-C chemokine receptor 2 (CCR2) [48, 49]. This contributes to T cell activation, hepatocyte death, and subsequent activation of hepatic stellate cells which are a major source of collagen-producing fibroblast expressing α-smooth muscle action (α-SMA) [50, 51]. In combination with regulatory T cells, TGFβ released from necrotic hepatocytes is involved in the pathogenesis of hepatic fibrosis by excessive accumulation of matrix proteins such as fibronectin, collagen 1, and protease inhibitors including TIMP, all of which promotes extracellular matrix production and decreases its degradation. Given effects of TGFβ on inflammatory infiltration during the development of NASH, increased collagen deposition could be observed as evident from the Masson’s trichrome staining [51–54]. In addition, negative inflammatory mediators such as SOCS1 and SOCS3 contribute to hepatic steatosis, inflammation, and fatty necrosis [32, 33]. In the current study, severely uncontrolled diabetic 38-week-old OLETF rats demonstrated significant increment of hepatic fat accumulation together with liver enzymes, oxidative stress, and cell death as demonstrated by serum ALT and AST concentrations, hepatic MDA levels, and number of TUNEL-positive cells. In addition, gene expression involved in proinflammatory cytokines (CD11b, F4/80, MCP1, and TNFα), negative inflammatory mediators (SOCS1 and SOCS3), and fibrosis-related proteins (TGFβ, collagen 1, and TIMP1) were significantly upregulated. In contrast to significantly increased inflammatory gene expression, an increasing trend but little or mild lobular inflammation and fibrosis stage were observed in H&E or Masson’s trichrome-stained liver tissues from 38-week-old OLETF rats. Therefore, we speculate that severely uncontrolled diabetic OLETF rats might have early stage of NASH. Six-week administration of rimonabant reversed the increases in hepatic TG deposition, serum levels of ALT and AST, lipid peroxidation, and cell death accompanied by the decreases in gene expression related to proinflammatory cytokines, negative inflammatory mediators, and fibrosis-related protein in a rat model of uncontrolled severe diabetes and early NASH.

Accumulating evidence suggests that two regulators of oxidative stress defense and inflammation, Nrf2 and AMPK, play a pivotal role in hepatic inflammation and lipid metabolism and the progression of NASH. High fat or methionine- and choline-deficient diets resulted in more severe NAFLD/NASH in Nrf2-null mice than in wild-type mice [20, 21]. Chronic Nrf2 activation attenuated hepatic fat accumulation by increasing antioxidant and detoxification ability and suppressing lipid synthesis in the liver [55–57]. These results demonstrate that Nrf2, a redox-sensitive transcription factor, has hepatic metabolic functions that have been linked to the pathogenesis of NAFLD/NASH. In the present study, we revealed that rimonabant administration increased gene expression of Nrf2 and its downstream genes such as NQO1, HO-1, GSTA, and TXNRD1. In addition, we found that CB1 receptor blockade significantly increased AMPK phosphorylation in the livers of OLETF rats. In a previous study, enhanced Nrf2 activity led to AMPK phosphorylation in the liver [22]. Several studies have demonstrated an inverse relationship between hepatic AMPK activity and fatty liver by reducing sterol-regulatory element-binding protein 1c (SREBP-1c) transcription factor and increasing phosphorylation and interaction with PGC1α [23, 58, 59]. Furthermore, AMPK activation reduces reactive oxidative stress and inflammation [24]. Given the close association between Nrf2 and AMPK activation and their effects on hepatic lipid metabolism and inflammation, Nrf2/AMPK activation could be a target for prevention or treatment of hepatic fat accumulation, inflammation, fibrosis, and death. Despite of critical findings illustrating the beneficial effects of CB1 receptor antagonist on hepatic fat inflammation, inflammation, and cell death at least partial involvement of increased Nrf2-AMPK pathway, animal model used in the present study did not develop NASH. To know exactly how rimonabant improves key features of fibrosis, further studies with NASH animal models are necessary to be executed.

In conclusion, the present study demonstrated that rimonabant administration ameliorates hepatic fat infiltration, inflammation, and cellular death, as well as mRNA expression of proinflammation and fibrosis genes in a rat model of severely uncontrolled diabetes. CB1 receptor blockade significantly increased expression involved in fatty acid oxidation, Nrf2, and its-related antioxidant response element mediated genes, and AMPK phosphorylation in liver tissues. Thus, our findings suggest the potential of pharmacological CB1 receptor blockade as a potential therapeutic tool in the progression of NASH.

## Abbreviations

ALT: alanine aminotransferase
AMPK: AMP-activated protein kinase
AST: aspartate transaminase
CB1: cannabinoid 1
CD11b: CD11 antigen-like family member b
CPT1α: carnitine palmitoyltransferase 1α
F4/80: EGF-like module-containing mucin-like hormone receptor-like 1
GSAT: glutathione S-transferase
HO-1: heme oxygenase 1
MCP1: CCR2 monocyte chemoattractant protein 1
Nrf2: nuclear factor erythroid 2-related factor 2
NQO1: NAD(P)H quinone oxidoreductase 1
OGTT: oral glucose tolerance test
OLETF: Otsuka Long-Evans Tokushima fatty
PPARα: peroxisome proliferator-activated receptor α
SOCS1: suppressor of cytokine signaling 1
SOCS3: suppressor of cytokine signaling 3
TGFβ: transforming growth factor β
TIMP1: tissue inhibitor of metalloproteinase 1
TNFα: tumor necrosis factor α
TUNEL: terminal deoxynucleotidyl transferase dUTP nick-end labeling
TXNRD1: thioredoxin reductase 1
